# Pro-oxidant status and matrix metalloproteinases in apical lesions and gingival crevicular fluid as potential biomarkers for asymptomatic apical periodontitis and endodontic treatment response

**DOI:** 10.1186/1476-9255-9-8

**Published:** 2012-03-21

**Authors:** Andrea Dezerega, Sonia Madrid, Verónica Mundi, María A Valenzuela, Mauricio Garrido, Rodolfo Paredes, Jocelyn García-Sesnich, Ana V Ortega, Jorge Gamonal, Marcela Hernández

**Affiliations:** 1 Laboratorio de Biología Periodontal, Facultad de Odontología, Avenida Sergio Livingstone 943, Comuna de Independencia, Santiago, Chile; 2 Escuela de Medicina Veterinaria, Facultad de Ecología y Recursos Naturales, Universidad Andres Bello, República 440 2° piso, Comuna de Santiago, Santiago, Chile; 3 Departmento de Bioquímica y Biología Molecular, Facultad de Ciencias Químicas y Farmacéuticas, Universidad de Chile, Avenida Vicuña Mackenna 20, Comuna de Providencia, Santiago, Chile; 4 Departmento de Patología, Facultad de Odontología, Universidad de Chile, Avenida Sergio Livingstone 943, Comuna de Independencia, Santiago, Chile

**Keywords:** Oxidant status, matrix metalloproteinases, apical periodontitis, apical lesions, gingival crevicular fluid

## Abstract

**Background:**

Oxidative stress and matrix metalloproteinases -9 and -2 are involved in periodontal breakdown, whereas gingival crevicular fluid has been reported to reflect apical status. The aim of this study was to characterize oxidant balance and activity levels of MMP -2 and -9 in apical lesions and healthy periodontal ligament; and second, to determine whether potential changes in oxidant balance were reflected in gingival crevicular fluid from asymptomatic apical periodontitis (AAP)-affected teeth at baseline and after endodontic treatment.

**Methods:**

Patients with clinical diagnosis of AAP and healthy volunteers having indication of tooth extraction were recruited. Apical lesions and healthy periodontal ligaments, respectively, were homogenized or processed to obtain histological tissue sections. Matrix metalloproteinase -9 and -2 levels and/or activity were analyzed by Immunowestern blot, zymography and consecutive densitometric analysis, and their tissue localization was confirmed by immunohistochemistry. A second group of patients with AAP and indication of endodontic treatment was recruited. Gingival crevicular fluid was extracted from AAP-affected teeth at baseline, after endodontic treatment and healthy contralateral teeth. Total oxidant and antioxidant status were determined in homogenized tissue and GCF samples. Statistical analysis was performed using STATA v10 software with unpaired t test, Mann-Whitney test and Spearman's correlation.

**Results:**

Activity of MMP-2 and MMP-9 along with oxidant status were higher in apical lesions (p < 0.05). Total oxidant status correlated positively with matrix metalloproteinase-2 and lesion size (p < 0.05). Gingival crevicular fluid showed significantly lower levels of total antioxidant status in diseased teeth at baseline compared to controls and endodontically-treated groups.

**Conclusions:**

Apical lesions display an oxidant imbalance along with increased activity of matrix metalloproteinase-2 and -9 and might contribute to AAP progression. Oxidant imbalance can also be reflected in GCF from AAP-affected teeth and was restored to normal levels after conservative endodontic treatment. These mediators might be useful as potential biomarkers for chair-side complementary diagnostic of apical status in GCF.

## Introduction

Asymptomatic apical periodontitis (AAP) corresponds to the inflammation and destruction of periradicular tissues caused by bacterial infection of dental pulp. It is the most common consequence of untreated dental caries and leads frequently to tooth loss. The hallmark of AAP is the presence of an apical lesion (AL) that results from destruction of hard and soft apical tissues [1,2].

The generation of reactive oxygen species (ROS), namely superoxide, hydroxyl and nitric oxide radicals, hydrogen peroxide and hypochlorous acid, represents an important pathogenic mechanism for diseases associated with phagocytic infiltration and bone resorption [3,4] as a host defense mechanisms against the invading pathogen [5]. Accordingly, neutrophils obtained from peripheral blood of AAP subjects have shown increased production of hydrogen peroxide and superoxide anion, which tend to normalize after surgical treatment [6,7]. Nevertheless, oxidant status in apical tissues remains unknown. As oxidant effects are additive, measuring the total oxidant (TOS) and antioxidant status (TAS) of a sample can provide a new and practical approach [8,9].

Matrix metalloproteinases (MMPs) are zinc and calcium-dependent endopeptidases that function at neutral pH. Fibrillar collagens are the major components of periodontal extracellular matrix. During periodontal homeostasis and pathologic conditions, they are cleaved into smaller fragments by collagenases (MMPs -1, -8, and -13) and further degraded by active gelatinases (MMPs -2 and -9) and other non specific tissue proteinases [10]. MMP-9 and MMP-2 have been identified through immunohistochemistry in experimentally-induced apical periodontitis in animal models where they were proposed to play a role in both, the initiation and progression of apical periodontitis [11,12]. Previous works have demonstrated increased mRNA expression levels of MMP-9 in apical granulomas in comparison to cysts [13,14], as well as MMP-9 activity levels in apical exudates from acute versus apical abscesses [15]. Furthermore, recent preliminary studies reported for the first time that gingival crevicular fluid (GCF) composition changes in AAP-affected teeth, showing increments in MMP-9 activity, frequency of detection of MMP-2 and total protein content in comparison to healthy controls [16,17]. Despite gelatinase activity has been reported in oral fluids during AAP, no previous determination of MMP-9 and -2 activities has been performed in apical lesions to support that these findings actually reflect apical status. Study of GCF particularly represents a novel approach in the search of biomarkers for apical periodontitis. In order to further understand whether changes in GCF reflect apical status, these changes should be demonstrated in both, apical lesions and GCF.

Additionally, new evidence of an oxidative regulation of MMP expression and activity in chronic periodontitis is emerging [18], but this association has never been reported in AAP, despite both diseases share common pathogenic mechanisms. Consequently, it is proposed that apical lesions present higher MMP-2 and MMP-9 activity compared to healthy PDL in association with oxidant status and that these changes can be reflected in GCF.

The aim of this study was first, to characterize and associate oxidant balance and the activity levels of MMP -2 and -9 in apical lesions and healthy periodontal ligament; and second, to determine whether potential changes in oxidant balance were reflected in GCF from AAP-affected teeth.

## Materials and methods

### Design: Analytic longitudinal study

#### Study subjects

Patients consulting at the clinics of diagnosis and endodontics, School of Dentistry, University of Chile were enrolled if they had clinical diagnosis of asymptomatic apical periodontitis (AAP) as previously described [19]. Diagnostic criteria included the presence of one or more apical lesions (compatible with an apical granuloma or cyst) detected by apical radiography (> 3 mm diameter) due to dental caries in teeth with clinical determination of non-vital pulp. The size of the radiographic apical lesion was registered as the average of vertical and horizontal diameters.

The teeth were selected if they had indication of tooth extraction or endodontic treatment. Exclusion criteria included marginal periodontal diseases defined by the absence of clinical attachment loss (≤ 2 mm), increased probing depths (≤ 3 mm) and bleeding on probing in less than 10% of the probing sites at examination; current smokers, systemic illness or previous antibiotics or non-steroid anti-inflammatory treatment during the 6-month period prior to the study. Healthy periodontal ligaments were also obtained from teeth with indication of extraction for orthodontic reasons from healthy donors as controls. All the protocols and procedures were approved by the Ethics Committee guidelines from University of Chile and in accordance with the ethical standards of the Declaration of Helsinki. The informed consent was obtained from all individuals. A minimal sample size of 10 patients in each group was determined with 90% power and a significance level of 5%.

### Tissue samples

**Figure 1:**
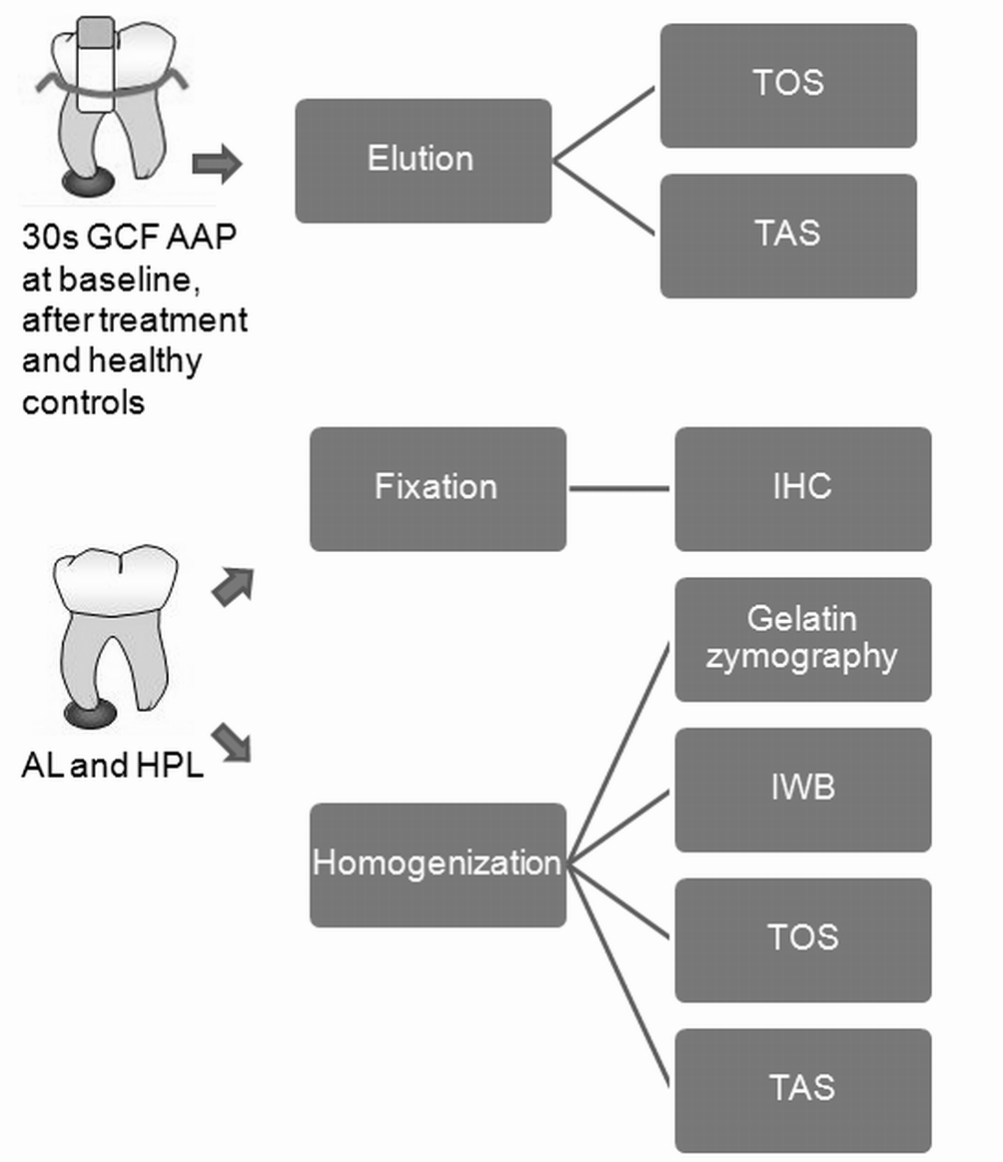
**Schematic representation of sample types and their respective analyses**. AAP: Asymptomatic apical periodontitis; TOS: Total oxidant status; TAS: Total antioxidant status AL: Apical lesions; HPL: Healthy periodontal ligament; IHC: Immunohistochemistry; IWB: Immunowestern blot;.

Apical lesions (AL) and healthy periodontal ligaments (PDL) were obtained from AAP and healthy subjects with indication of teeth extraction for subsequent tissue homogenization (AL, n = 10; PDL, n = 13). A second group of tissue samples were fixed in buffered formaldehyde at 4% and included in paraffin for routine processing, diagnosis of apical granuloma (n = 4) or healthy periodontal ligament (n = 4), and immunohistochemistry (Figure 1). Mean age for apical periodontitis group was 50.1 ± 17.5 years old and 23.2 ± 3.8 years old for control group. Among them, 5 and 8 corresponded to females in each group, respectively.

Tissue samples for homogenization procedures were weighted and protein extracts were prepared by an automated homogenizer in 50 mM Tris-HCl pH 7.5, 0.2 M NaCl, 5 mM CaCl_2 _and 0.01%Triton X-100 buffer adding EDTA-free proteinase inhibitor cocktail (Roche Diagnostics GmbH) in a constant ratio of 10 μL of buffer per mg of weighted tissue, centrifuged at 13000 × g for 6 min. at 4°C; the pellet was resuspended in the same buffer and kept under -80° until further analysis.

#### Gingival crevicular fluid samples

GCF samples were obtained from patients having indication of conservative endodontic treatment from around affected teeth (N = 16) at baseline, one week after completion of the endodontic treatment, and from controls corresponding to healthy contra lateral tooth in each subject (Figure 1). The mean age was 39.5 years old and 12 corresponded to females.

After isolating the tooth with a cotton roll, the crevicular site was gently dried with an air syringe. GCF was collected with standard commercial paper strips (ProFlow, Amityville, New York, USA). Strips were placed into the sulcus until mild resistance was sensed and left in place for 30s. Strips contaminated by saliva or blood were excluded.

After GCF collection, strips were placed in microtubes and kept at -20°C. GCF was extracted by centrifugation at 9000 × g for 6 min in a final volume of 5 μL of elution buffer containing 50 mM Tris-HCl pH 7.5, 0.2 M NaCl, 5 mM CaCl_2 _and 0.01%Triton X-100, adding EDTA-free proteinase inhibitor cocktail (Roche Diagnostics GmbH) and samples were stored at -20°C until further analysis.

### Sample analysis

#### Immunohistochemistry

To confirm tissue localization of MMPs -2 and -9, tissue sections of 6 micrometers were cut, deparaffinized and endogenous peroxidase activity was quenched by incubation in 10% hydrogen peroxide. Antigen retrieval was done with Proteinase K following manufacturer's recommendations (Novocastra^®^, Lab. Novo, Newcastle, UK) and unspecific blocking was performed with 2.5% horse serum for 10 min. (Kit ABC Universal, RTU Vectastain^® ^Kit for laboratory use, Burlingame, CA, USA.). Respective monoclonal primary antibodies against human MMP-2 or MMP-9 (R&D Systems, Inc^®^, Minneapolis, MN, USA), were incubated overnight in 1:20 and 1:10 dilutions, respectively and rinsed. The immunostainings were performed with Vectastatin Elite ABC kit (Vector Laboratories, Burlingame, CA), using anti-mouse biotinylated secondary antibody, revealed with DAB (Peroxidase Sustrate Kit, Zymed Labs INC^®^, San Francisco, CA, USA), counterstained with Mayer's hematoxylin (Merck KGaA, Darmstadt, Germany) and mounted. Slides were examined using an optical microscope (Zeiss, Axiostar Plus^®^, NY, USA) and representative images were acquired using a digital camera (Canon, Powershot A640, Tokyo, Japan). Positive and negative controls were processed with each series.

#### Gelatin zymography

Gelatinase activity was assessed in tissue homogenates. Aliquots were run under non-reducing denaturing conditions in 10% SDS/PAGE gels containing 1 mg/mL gelatin as substrate, soaked twice in 2.5% Triton X-100 and incubated in developing buffer (20 mM Tris pH 7.4 and 5 mM CaCl2) during 17 h, stained with Coomassie Brilliant Blue R-250 and destained with 10% acetic acid and 20% methanol solution. To measure gelatinolytic activities, densitometric analysis was performed using UNSCAN-IT gel automated digitizing system V4.1 software (Silk Scientific Corporation, Orem, UT, USA) and results were expressed as arbitrary densitometric units/mg of tissue (au/mg).

#### Identification of MMP-2 and MMP-9 by immunoimmunowestern blots

To identify MMP-2 and MMP-9 immunoreactivities and confirm the identity of gelatinolytic bands, aliquots of tissue homogenates were run under reducing conditions into 10% SDS/PAGE gels and transferred to nitrocellulose membranes. The membrane was blocked with 3% skim milk in Tris Buffered Saline - Tween 0.1% (TBS-T) for 1 hour. Primary monoclonal antibodies against MMP-2 and MMP-9 (R&D Systems, USA) in 1:200 and 1:100 dilutions respectively were added and the membrane was incubated overnight. The membrane was washed with TBS-T, incubated for 1 hour with HRP-conjugated secondary antibody, washed again and detection was performed with an enhanced chemiluminiscence (ECL) detection kit (Femto, Pierce, Rockford, USA).

#### Total Oxidant Status (TOS)

TOS levels in tissue homogenates and GCF eluates were determined by the measurement method previously described [8]. The assay was calibrated with hydrogen peroxide (H_2_O_2_). Briefly, 225 μl Reagent 1 (150 μM xylenol orange, 140 mM NaCl and 1.35 M glycerol in 25 mM H_2_SO_4 _solution, pH 1.75) was mixed with 35 μl of each respective sample and the absorbance at 560 nm was read with a spectrophotometer as each sample blank. Then, 11 μl Reagent 2 (5 mM ferrous ion and 10 mM o-dianisidine in 25 mM H_2_SO_4 _solution) was added and the absorbance was read again at 560 nm after 3 min. The results were expressed as μmol H_2_O_2 _equiv/L. per mg of tissue homogenates and per 30 s. of GCF sampling.

#### Total Antioxidant status (TAS)

Tissue homogenates and GCF eluates were measured by a Total Antioxidant Status commercial kit (Randox Laboratories, Ardmore, UK) as previously performed [20]. Briefly, metmyoglobin is turned into ferrymyoglobin in the presence of iron. Addition of Randox reagent di-(3-ethylbenzthiazoline sulphonate) (ATBS^®^) results in the formation of a blue-green coloured radical that could be detected at 630 nm. The inhibition of radical formation by the antioxidants from the sample was calculated in mM quantities of ATBS^® ^disappearance and expressed per mg of tissue for homogenates and per 30 s. of GCF sampling.

### Statistical analysis

Statistical analysis was performed using STATA v10 software (StataCorp, Collage Station, TX, USA). MMPs, TOS and TAS comparisons between AL and control tissue groups were analyzed by unpaired t test and Mann-Whitney test, depending whether data distribution was normal or not and Spearman's correlation. TOS and TAS comparisons in GCF between AAP versus contralateral control teeth and baseline versus post-treatment determinations were performed using paired-t test. Statistical significance was considered if p < 0.05.

## Results

### Apical lesions and healthy periodontal ligament

**Figure 2:**
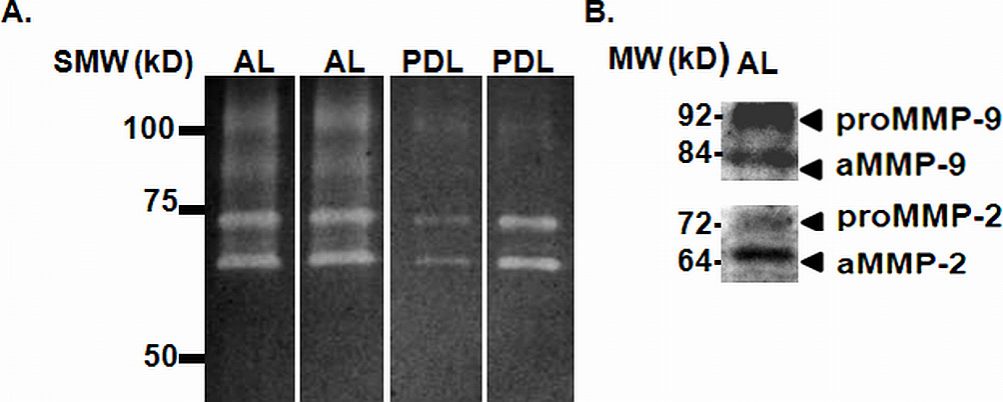
**MMP-9 and MMP-2 enzymatic forms in apical lesions and healthy periodontal ligament**. Representative MMP-9 and MMP-2 enzymatic forms identified by gelatin zymography. B. MMP-9 and MMP-2 enzymatic forms identified by immunowestern blot. SMW: Standard of molecular weight (kD); AL: Apical lesions; PDL: Healthy periodontal ligament; proMMP-9 and proMMP-2: Proenzyme forms; aMMP-9 and aMMP-2: Active enzyme forms.

**Figure 3:**
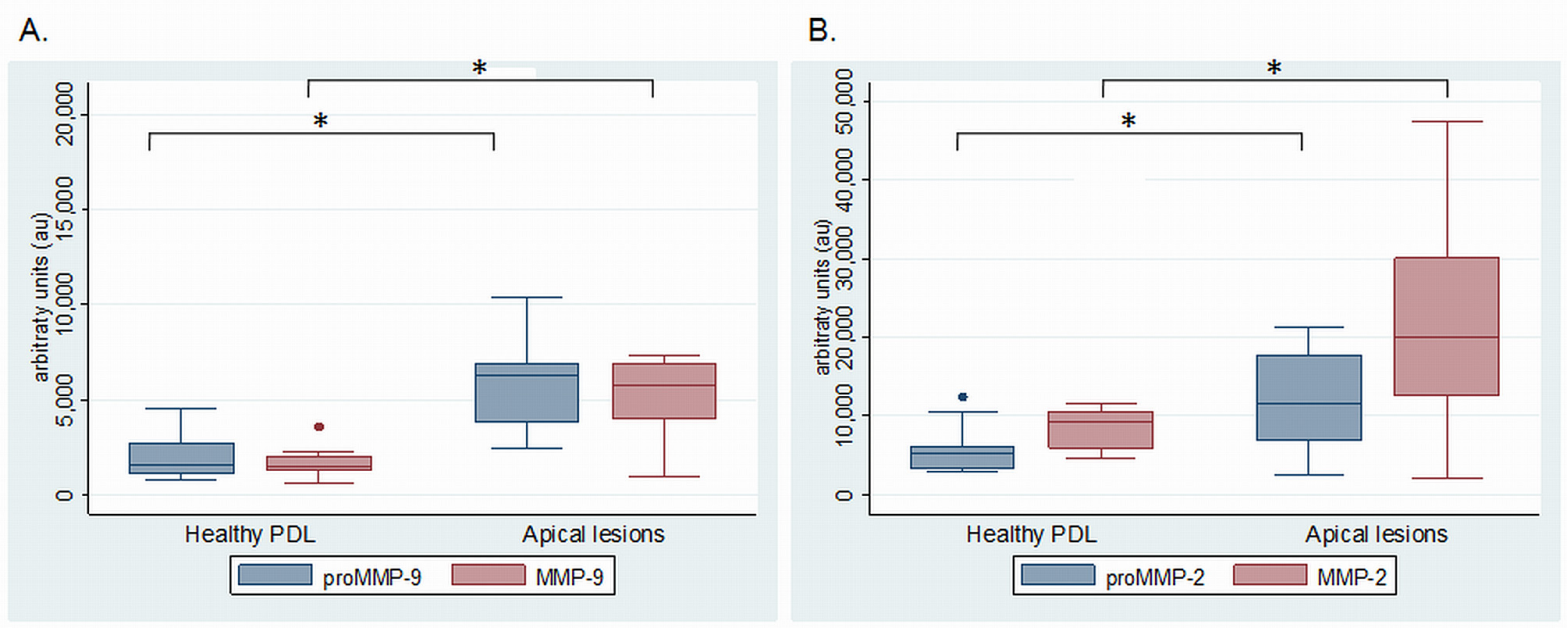
**Activity levels of MMP-9 (A) and MMP-2 (B) in apical lesions and healthy periodontal ligament**. PDL: Healthy periodontal ligament; proMMP-9 and proMMP-2: proenzyme forms; aMMP-9 and aMMP-2: Active enzyme forms. *p < 0.05.

MMP-9 and MMP-2 enzymatic forms were detected by gelatin zymography in all tissue homogenates from apical lesions and healthy periodontal ligaments (Figure 2A). Immunoreactivities for MMP-9 and MMP-2 proenzyme and active forms were confirmed by Immunowestern blot (Figure 2B). Significantly higher gelatinolytic activity corresponding to active enzyme and proforms of MMP-9 were found in apical lesions compared to healthy PDL (p = 0.0004 and p = 0.001, respectively). Similarly, MMP-2 bands corresponding to active and proenzyme forms were significantly higher in diseased group (p = 0.018 and p = 0.009, respectively) (Figure 3).

**Figure 4:**
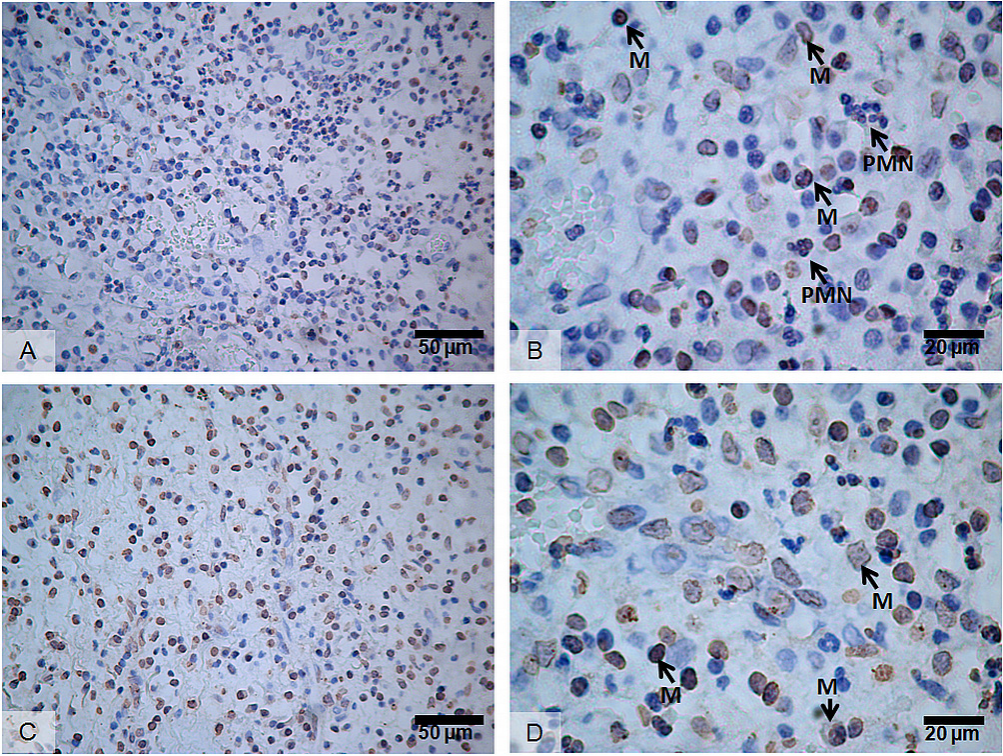
**Immunolocalization of MMP-9 and MMP-2 in apical granulomas**. A-B: MMP-9; C-D: MMP-2; M: Mononuclear inflammatory cells; PMN: Neutrophils.

Immunopositive cells for MMP-9 and MMP-2 in apical granulomas were mainly localized to inflammatory infiltrates (Figure 4, A-D). MMP-9 immunopositive cells were identified according to cell morphology as neutrophils (PMNs) and mononuclear cells (Figure 4B). Similarly, MMP-2 was immunolocalized to mononuclear cells (Figure 4D).

TOS and TAS were higher in apical lesions compared to healthy periodontal ligament, but differences were significant only for TOS (p = 0.006 and p = 0.06, respectively; Table 1).

**Table 1:** TOS and TAS levels in tissue samples and GCF from asymptomatic apical periodontitis and controls.

	AAP	Controls	p	AAP BL	AAP AT	p
Tissue TOS (μmol H_2_O_2 _equiv)	29.33 ± 3.52	25.60 ± 2.74	**0.006**	---	---	---

Tissue TAS (mM)	1.36 (0.85)	0.42 (2.19)	0.06	---	----	---

GCF TOS (μmol H_2_O_2 _equiv)	2.44 ± 1.38	2.50 ± 2.33	0.46	2.81 ± 2.02	3.02 ± 2.17	0.39

GCF TAS (mM)	0.93 ± 0.68	1.34 ± 0.75	**0.03**	0.74 ± 0.62	1.20 ± 0.80	**0.02**

Values expressed as means ± standard deviation or medians (interquartile range). AAP: Asymptomatic apical periodontitis; BL: baseline; AT: after treatment.

Correlation analysis of all tissue homogenates demonstrated that TOS correlated positively with active MMP-2 (r = 0.58, p = 0.008). Active MMP-2 also correlated with proMMP-9 (r = 0.6, p = 0.005), as well as active MMP-9 (r = 0.45, p = 0.047), but the later was borderline significant. When the aforementioned associations were analyzed separately (Table 2), TOS and active MMP-2 still displayed a tendency to a positive correlation (r = 0.7, p = 0.058) in apical lesions, whereas TOS and TAS were inversely correlated (r = -0.8, p = 0.009). TOS also correlated positively with apical lesion size (r = 0.6, p = 0.02). No correlations for TOS were found in healthy PDL group.

**Table 2:** Spearman's correlation matrix between MMPs and oxidative balance in apical lesions and healthy periodontal ligament.

	proMMP-9		aMMP-9		proMMP-2		aMMP-2		TOS		TAS	
	
	AL	PDL	AL	PDL	AL	PDL	AL	PDL	AL	PDL	AL	PDL
proMMP-9	1	1	---	---	---	---	---	---	---	---	---	---

aMMP-9	-0.3	-0.2	1	1	---	---	---	---	---	---	---	---

proMMP-2	0.2	0.6	0.1	-0.4	1	1	---	---	---	---	---	---

aMMP-2	0.28	0.4	0.3	-0.2	0.4	0.3	1	1	---	---	---	---

TOS	0.3	-0.6	-0.3	0.4	0.6	-0.4	0.7	0.03	1	1	---	---

TAS	-0.01	-0.3	0.1	-0.6	-0.6	0.2	-0.6	0.17	-0.8*	0.05	1	1

Lesion size	0.03	---	0.38	---	0.4	---	0.7	---	**0.6^#^**	---	-0.6	---

AL: apical lesions, n = 9; PDL periodontal ligament, n = 11. Each box shows respective rho. Bolds show significant correlations; *p = 0.009; #p = 0.02.

Because the mean age varied considerably among donors of apical tissue samples (apical lesions and healthy PDL), the existence of a potential association between age and the levels of the studied mediators was assessed by Spearman's correlation. No association was found between TOS, MMP-9 or MMP-2 and age (p > 0.05); whereas TAS and age displayed a positive correlation of borderline significance in the diseased group (r = 0.68, p = 0.044).

### GCF

Analysis of TOS and TAS levels in GCF from AAP subjects (Table 1) demonstrated no differences in TOS levels between AAP and respective contralateral control samples, as well as AAP at baseline and after conservative endodontic treatment (p > 0.05). TAS on the other hand, was found to be significantly lower in AAP samples in comparison to controls (p = 0.03). TAS levels after treatment increased significantly compared to baseline, reaching levels similar to control samples (p = 0.02).

## Discussion

Periapical lesions result from the dynamic encounter between microbial factors and host immune response in subjects with clinical diagnosis of asymptomatic apical periodontitis. Both, granulomas and radicular cysts, are considered to represent two different stages of development of the same inflammatory process and are characterized by leukocyte infiltration. These cells represent important regulators of extracellular matrix turnover and destruction, representing the major source of bone-resorbing mediators, including reactive oxygen species and MMPs [5,6,16].

The results of the current study support that an oxidative imbalance along with increments in MMP-9 and MMP-2 levels and activity might play a role in the development and/or progression of apical lesions. Furthermore, oxidative imbalance at the expense of reduced antioxidant levels was also reflected in GCF of AAP teeth, in comparison to healthy controls and endodontically treated teeth.

Leukocytes and resident fibroblasts from periodontal tissues are known to secrete MMPs, including MMPs -1, -2, -3, -8, -9 and -13 [21-24]. During the last few years, MMPs -2 and -9 have shown to be over expressed at the mRNA level in apical granulomas [14,15] and higher activity levels have been reported in apical exudates from acute apical abscesses, as well as in GCF from teeth affected with asymptomatic apical periodontitis versus healthy teeth [16]. The results of the current study complement the previous findings reported in GCF, showing significantly higher activity levels of both, proenzymes and active forms of MMP-9 and MMP-2 in apical lesions when compared to healthy periodontal ligaments. Additionally, tissue localization of MMP-9 and MMP-2 was confirmed in apical granulomas by imunohistochemistry [13]. The study of GCF as a source of potential biomarkers for AAP represents a novel approach that needs further validation. Overall, these data support that changes in MMP activity in GCF are similar as those for apical lesions.

Previous works support that MMP -2, MMP-9 and MMP-13 play important roles in both, the initiation and progression of inflammatory bone resorption and soft periodontal tissue breakdown during pathological processes, including chronic periodontitis [21,25-27]. Under inflammatory conditions, bone resorptive mediators, like interleukin-1 and prostaglandin E2, induce a marked expression of RANKL and MMPs, such as MMP-13, -3 and -2 by osteoblasts and MMP-9 by activated osteoclasts and leukocytes [1,28]. Additionally, MMP-9 activity is thought to act over preosteoclast recruitment and migration [29].

High levels of oxidants in tissues perturb the normal redox balance and shift cells into a state of oxidative stress [30-32]. In the current study, we evidenced an oxidant imbalance in favor to ROS in both, apical lesions and GCF from AAP teeth versus healthy controls and endodontically-treated teeth. Evidence has led ROS to become increasingly implicated in the damage of extracellular matrix components from connective tissue incurred during inflammatory diseases [33]. At the cellular level, ROS activate redox-sensitive transcription factors, including NF-κB and AP-1, thereby causing indirect tissue damage and exacerbation of inflammation [9]. A positive correlation between TOS and the bone resorptive area and a negative correlation between TOS and TAS was found in apical lesions, but not in healthy PDL. An imbalance in favor to ROS might stimulate apical bone loss. Recent evidence has shown that ROS might play an osteolytic role by suppressing bone formation through inhibition of osteoblastic differentiation, and by stimulating osteoclast differentiation and bone resorption [34] through induction of receptor activator of NFκB ligand (RANKL). Additionally, higher MMP-2 expression and activation in response to ROS has been reported [3,35,36], evidencing a key link between ROS production, MMP-mediated proteolysis and bone resorption, which could play a central role in the progression of apical lesions. In line with these findings, an association between TOS and active MMP-2 was found in the current study, suggesting that pro-oxidant status and MMP-mediated proteolysis might be cooperative in nature during AAP progression. In support of this, an association between active MMP-8 and myeloperoxidase has recently been reported during chronic periodontitis progression [18].

Additionally, based on the fact that MMP-9 can induce proMMP-2 activation *in vitro *[37,38], the finding of a positive correlation between active MMP-9 and active MMP-2 suggests that this activation mechanism might also occur in vivo. A limitation of this study was the age difference found between AAP groups and their respective controls. Healthy erupted teeth are obtained for orthodontic purposes mostly in young patients. Nevertheless, a lack of association between age, TOS and MMPs supports that the differences found resulted from shifting of apical status. A deeper knowledge of the mechanisms involved in destruction of apical periodontium will contribute to the development of improved methods of diagnosis, treatment and follow-up for side-diagnostic tools.

GCF represents a simple, non-invasive and useful tool in monitoring inflammation and treatment response in marginal periodontal diseases [39,40], but it has rarely been studied in apical periodontitis. MMP activity or active forms in GCF, particularly of MMP--13, MMP-9 and MMP-8, have previously been associated with progression and/or severity of marginal chronic periodontitis [26,41]. Recently, changes in GCF composition, involving higher gelatinase activity [16] and protein concentration, were reported in AAP in comparison to healthy controls [17]. These studies provide preliminary evidence supporting that GCF might reflect the health status of the apical tissues, as well as apical disease progression. In line with these reports, the present study shows the occurrence of an oxidant imbalance in apical lesions and also in GCF from AAP teeth, whereas endodontic treatment appears to restore antioxidant status to its normal levels. Considering that treatment outcome is difficult to predict based solely upon clinical and radiographic criteria and classical samples for endodontic purposes are invasive, GCF measurement of gelatinase activities along with the oxidant balance might represent a useful side- diagnostic tool, but further studies are needed to confirm whether GCF can reflect apical inflammation, its resolution and apical healing.

## Conclusions

Inflammatory apical lesions were characterized by pro-oxidant status and elevated MMPs -2 and -9 activity, supporting their involvement in the pathogenesis of apical periodontitis. Oxidant imbalance was also reflected in GCF from affected teeth and was restored after conservative endodontic treatment. These mediators might be useful as potential biomarkers for chair-side complementary diagnostic of apical status in GCF.

## Competing interests

The authors declare that they have no competing interests.

## Authors' contributions

DA, GM, OAV, GJ: Contributed to study design and funding acquisition, enrolment of study subjects, performance of clinical evaluation and treatments, data interpretation and manuscript preparation; VMA, PR, G-S J, MS, MV: Contribution of study design, sample preparation, biochemical and immunobiochemical analyses, data interpretation and manuscript preparation; HM: Study conception and design, funding acquirement, supervision of laboratory procedures, data analysis and interpretation, manuscript writing and preparation. All authors read and approved the final manuscript.
